# Potential therapeutic effects of an ayahuasca-inspired N,N-DMT and harmine formulation: a controlled trial in healthy subjects

**DOI:** 10.3389/fpsyt.2023.1302559

**Published:** 2024-01-08

**Authors:** Helena D. Aicher, Michael J. Mueller, Dario A. Dornbierer, Dila Suay, Claudius Elsner, Ilhui Wicki, Daniel Meling, Luzia Caflisch, Alexandra Hempe, Camilla Steinhart, Jovin Mueller, Robin Von Rotz, Birgit Kleim, Milan Scheidegger

**Affiliations:** ^1^Psychedelic Research and Therapy Development, Department of Psychiatry, Psychotherapy and Psychosomatics, Psychiatric University Hospital Zurich, University of Zurich, Zurich, Switzerland; ^2^Department of Psychology, University of Zurich, Zurich, Switzerland; ^3^Neuroscience Center Zurich, University of Zurich and ETH, Zurich, Switzerland; ^4^Department of Health Science and Technology, ETH, Zurich, Switzerland; ^5^Institute of Pharmacology and Toxicology, University of Zurich, Zurich, Switzerland; ^6^Department of Forensic Pharmacology and Toxicology, University of Zurich, Zurich, Switzerland; ^7^MoMiLab, IMT School for Advanced Studies Luca, Lucca, Italy; ^8^Department of Psychosomatic Medicine and Psychotherapy, Medical Center–University of Freiburg, Faculty of Medicine, University of Freiburg, Freiburg, Germany; ^9^Biopsychology, Department of Psychology, TUD Dresden University of Technology, Dresden, Germany; ^10^Experimental Psychopathology and Psychotherapy, Department of Psychiatry, Psychotherapy and Psychosomatics, Psychiatric University Hospital Zurich, University of Zurich, Zurich, Switzerland

**Keywords:** ayahuasca, ayahuasca analog, DMT, harmine, therapeutic potential, psychological processes, psychometrics, subjective effects

## Abstract

**Background:**

There is growing scientific evidence for the therapeutic benefits of the Amazonian plant-based psychedelic “ayahuasca” for neuropsychiatric disorders such as depression and anxiety. However, there are certain challenges when incorporating botanical ayahuasca into biomedical research and clinical therapy environments. Formulations inspired by ayahuasca, which contain specific and standardized active components, are a potential remedy.

**Methods:**

We investigated subjective acute and persisting effects of a novel formulation containing the reversible monoamine oxidase inhibitor harmine (orodispersible tablet containing 100 mg MAO-I) and N,N-dimethyltryptamine (incremental intranasal dosing of up to 100 mg DMT), compared with two other conditions, namely harmine alone and placebo, in a crossover RCT in 31 healthy male subjects.

**Results:**

DMT + harmine, but not harmine alone, induced a psychedelic experience assessed with the 5D-ASC rating scale [global score: *F*(2,60) = 80.21, *p* < 0.001] and acute experience sampling items over time, characterized by psychological insights [PIQ, *F*(2,58.5) = 28.514, *p* < 0.001], emotional breakthroughs [EBI, *F*(2,60) = 26.509, *p* < 0.001], and low scores on the challenging experience questionnaire [CEQ, *F*(2,60) = 12.84, *p* < 0.001]. Participants attributed personal and spiritual significance to the experience (GSR) with mainly positive persisting effects (PEQ) at 1- and 4-months follow-up. Acute drug effects correlated positively with persisting effects. We found no changes in trait measures of personality, psychological flexibility, or general well-being, and no increases in psychopathology (SCL-90-R) were reported.

**Discussion and Conclusion:**

Our results suggest that the experience induced by the standardized DMT + harmine formulation induces a phenomenologically rich psychedelic experience, demonstrates good psychological safety and tolerability, is well tolerated, and induces beneficial psychological processes that could possibly support psychotherapy. Further studies are required to investigate the psychotherapeutic potential in patients.

## 1 Introduction

Scientific interest in the N,N-dimethyltryptamine (DMT) containing Amazonian psychedelic “ayahuasca” has increased due to its potential therapeutic benefits (1–3) and its increasingly globalized use (4). This plant-based brew has been used by Indigenous cultures for centuries for ceremonial and medicinal purposes (5). Ayahuasca contains the vine *Banisteriopsis caapi* and other ingredients such as *Psychotria viridis* or *Diplopterys cabrerana* (6). The vine contains a variety of 𝛽-carbolines, some of which are selective reversible monoamine oxidase inhibitors (MAO-Is; e.g., harmine and harmaline), or serotonin reuptake inhibitors (e.g., tetrahydroharmaline) (7). The MAO-Is prevent the degradation of the N,N-dimethyltryptamine (DMT) contained in the *Psychotria viridis* leaves or other additional plants, thereby DMT becomes orally bioavailable (7–9). DMT is a structural analog of serotonin and has agonistic properties at a variety of serotonin receptors (mainly 5-HT2A, 5-HT2C, 5-HT1A), but also glutamate, dopamine, acetylcholine, sigma-1, and trace amine-associated receptors (9–12), similar to other psychedelics such as psilocybin or lysergic acid diethylamide (LSD) (13).

In contrast to the rapid onset and heightened experiential intensity of intravenous or inhaled DMT administration (14–18), the subjective effects of orally ingested DMT in ayahuasca preparations exhibit a gradual onset and dissipation over a period of 4 to 6 h (10, 11). The ayahuasca plant composition induces psychedelic effects, including alterations in perception of the self and the world, changes in mood and cognition, visionary experiences and bodily sensations (10, 19, 20).

Transient introspective states induced by ayahuasca are characterized by increased awareness, introspection and self-reflection, dream-like visions, recollection of personal memories, and intensified emotions (10, 11, 20), potentially leading to deep psychological and existential insights (21), which might promote the efficacy of psychotherapeutic interventions (22–25). Psychological insights refer to the understanding of own potentially maladaptive, but also goal oriented relational, behavioral, and cognitive patterns (26). Similarly, emotional breakthrough experiences describe moments of emotional clarification and uncovering of repressed affect (27). This combination of psychological insights and emotional breakthrough experiences represents an essential aspect of transformative psychotherapeutic processes. Indeed, definitions of catharsis–derived from the Greek word for cleansing or purification, which are terms often used in the context of ayahuasca rituals – emphasize two components: emotional and cognitive aspects of catharsis (28). It has been proposed that the development of insights following emotional catharsis is crucial for sustainable improvement in psychosomatic functioning, while purely cognitive insights could result in increased psycho-physiological tension (29). Therefore, psychological insights and emotional breakthrough experiences represent potentially important target mechanisms for the exploration of the therapeutic effects of these substances. In our study, the Psychological Insights Questionnaire (PIQ) and the Emotional Breakthrough Inventory (EBI) were used to assess these psychological processes.

Indeed, converging evidence from preclinical and human studies suggest that oral formulations of DMT such as ayahuasca show potential in reducing anxiety, depressive symptoms, addictive behaviors, and posttraumatic distress (1–3, 30–35). In small clinical pilot studies, ayahuasca has been shown to alleviate symptoms of depression and anxiety (30). Several studies, including one RCT, have highlighted a similar rapid onset and sustained antidepressant effect after a single ingestion in patients diagnosed with treatment-resistant depression (3, 36). Hence, ayahuasca may be particularly well suited for the systemic treatment of complex affective diseases, where currently available antidepressants have often show limited efficacy, a prolonged onset of action, and less favorable side effect profiles due to daily administration (25, 37, 38).

Compared to ayahuasca, there are substantial shortcomings of using other psychedelic compounds for psychotherapy. LSD, for example, has a significantly longer duration of action (~10 h). A single dose of ayahuasca with an overall duration of 2–4 h is more amenable for clinical applications (10). Both LSD and psilocybin show rapid tolerance at serotonergic receptors (13, 39), which can be a disadvantage for clinical application (40) and makes them less suited for repeated clinical dosing regimens. Although repeated administration of ketamine was shown to sustain antidepressant effects (41, 42), chronic ketamine use puts patients at risk due to its addictive properties and some long-term toxicity (43). In contrast, ayahuasca does not induce tolerance even after repeated administration (44, 45), is considered safe, and shows no addictive abuse potential (44, 46, 47). This underscores the experimental utility of DMT for the investigation of brain serotonin function and its role in emotional processing and social cognition as well as its therapeutic potential in the context of psychedelic-assisted (psycho)therapy.

However, there are some challenges in the clinical implementation of ayahuasca into the Western medical system. First of all, there are physiological side effects, including nausea and vomiting (48). While vomiting is considered a purification and one of the healing mechanisms of ayahuasca in traditional contexts (49), in a biomedical clinical setting, vomiting is seen as the body’s reaction to the toxins contained in ayahuasca (e.g., harmaline) and is regarded an undesired and adverse side effect. Additionally, ayahuasca brew batches have varying levels and ratios of alkaloids due to different recipes including differences in plant combinations and ratios, variations in cooking duration and method, and naturally varying alkaloid levels in the plants used, which complicates the creation of standardized dosing protocols (6, 50). Furthermore, bioavailability of the psychoactive compound DMT varies greatly between and within individuals due to the excessive metabolic first-pass degradation by the MAO enzyme associated with oral ingestion of ayahuasca, which potentially leads to unexpected intensities, overwhelming experiences, and thereby increases psychological risks (48). Consequently, targeted medication in the sense of standardizing pharmaceutical treatment is made difficult.

Additional significant cultural, sociological, and environmental factors make the use of traditional ayahuasca in the Western medical system questionable. In Indigenous tribes, ayahuasca refers to a holistic healing system and a worldview that cannot be directly transposed to our cultural context (51), also due to the inherent risk of cultural appropriation (52). The overuse of plants contained in ayahuasca is another consequence of ayahuasca tourism and plant export, that come along with the globalization of ayahuasca (53).

Extending upon the intricate considerations surrounding cultural, social, and ecological aspects mentioned above, our efforts to address this multifaceted challenge has led to the development of an innovative ayahuasca-inspired formulation containing DMT and harmine, two of the main active compounds of ayahuasca. Improving the tolerability with a more controllable delivery mechanism poses significant advantages for clinical translation into the context of psychedelic-assisted therapy. Furthermore, we implemented an incremental dosing of DMT, which we hypothesized would allow for better controllability and thereby represent a potentially more patient-oriented administration regimen. We conducted a randomized controlled trial in healthy participants to compare acute and post-acute effects of combined DMT + harmine (DMT/HAR) with harmine only (HAR) and placebo (PLA) and potential persisting effects of the overall study experience, with a focus on psychological mechanisms of action including psychological safety and tolerability assessments. Based on the findings of Turner & Merlis (54) that intranasal DMT was pharmacologically inactive, we did not include a DMT only condition. Harmine as a MAO-I was chosen, because it’s a major psychoactive molecule of the ayahuasca brew with a favorable side effect profile, e.g., lower toxicity compared to harmaline, and because it’s selective and reversible (55).

We hypothesized that combined DMT + harmine, but not harmine alone, would be characterized by psychedelic phenomena as assessed with the Altered States of Consciousness (ASC) questionnaire, but with only low levels of anxiety. Furthermore, we hypothesized the DMT + harmine formulation, but not harmine alone, to elicit psychological insights (assessed with the Psychological Insights Questionnaire; PIQ) and emotional breakthroughs (assessed with the Emotional Breakthrough Inventory; EBI). We included the Challenging Experience Questionnaire (CEQ) and the Symptom Checklis-90-Revised (SCL-90-R) as measures of psychological safety and tolerability. In our dose-finding pilot study (56) comparing different doses and administrations routes, we developed optimized delivery mechanism and the incremental dosing. Due to these optimized procedures, in the present study we hypothesized that only low levels of challenging experiences (CEQ), however stronger in the DMT/HAR compared to HAR and PLA, but not different in the HAR compared to the PLA condition, would be reported. No increases in psychopathological symptoms (SCL-09-R) following the study experience were expected. We further hypothesized to find primarily positive persisting effects (assessed with the Persisting Effects Questionnaire; PEQ) and further that these would be associated with the acute experience. We hypothesized that trait measures would changes favorably (increase in psychological flexibility, AAQ2; cognitive flexibility, CFI; nature relatedness, NR6; wellbeing, WHO-5; big 5 openness, decrease in neuroticism). Moreover, we hypothesized that participants would attribute significance to the experience (Griffiths significance ratings; GSR).

## 2 Materials and methods

### 2.1 Participants and permissions

Thirty-seven healthy male participants were recruited, six dropped out (four before the first intervention day, two after the first intervention day). Thirty-one participants (age 25.39 ± 4.21 years) completed all 3 intervention days. Following criteria were required for inclusion: (i) male sex in order to avoid the potential impact of menstrual cycle on blood chemistry, (ii) age 20–40 years, (iii) BMI 18.5–30 kg/m2, (iv) no current or previous history of somatic, neurological, or psychiatric disorder, (v) no family history of psychosis, bipolar, or other severe psychiatric disorders, (vi) no acute or chronic medication intake that could interact with the study drug, (vii) no current or regular drug intake (not more than 15 lifetime psychedelic experiences). Participants were required to abstain from caffeine on test days, as well as alcohol the day before; they had to refrain from using psychoactive substances or other medications for 2 weeks prior to the test days and throughout the study period. A sample description can be found in the Supplementary material (detailed demographics, motivation to participate, previous drug experience; Supplementary Tables S1–S4). Participants received monetary compensation (320.- CHF total, or 60.- per completed intervention day).

The study was approved by the Cantonal Ethics Committee of the Canton of Zürich (Basec-Nr. 2018–01385) and Swiss Federal Office of Public Health [BAG-Nr. (AB)-8/5-BetmG-2019/008014] All participants provided written informed consent according to the declaration of Helsinki.

### 2.2 Study design and procedure

**Figure 1:**
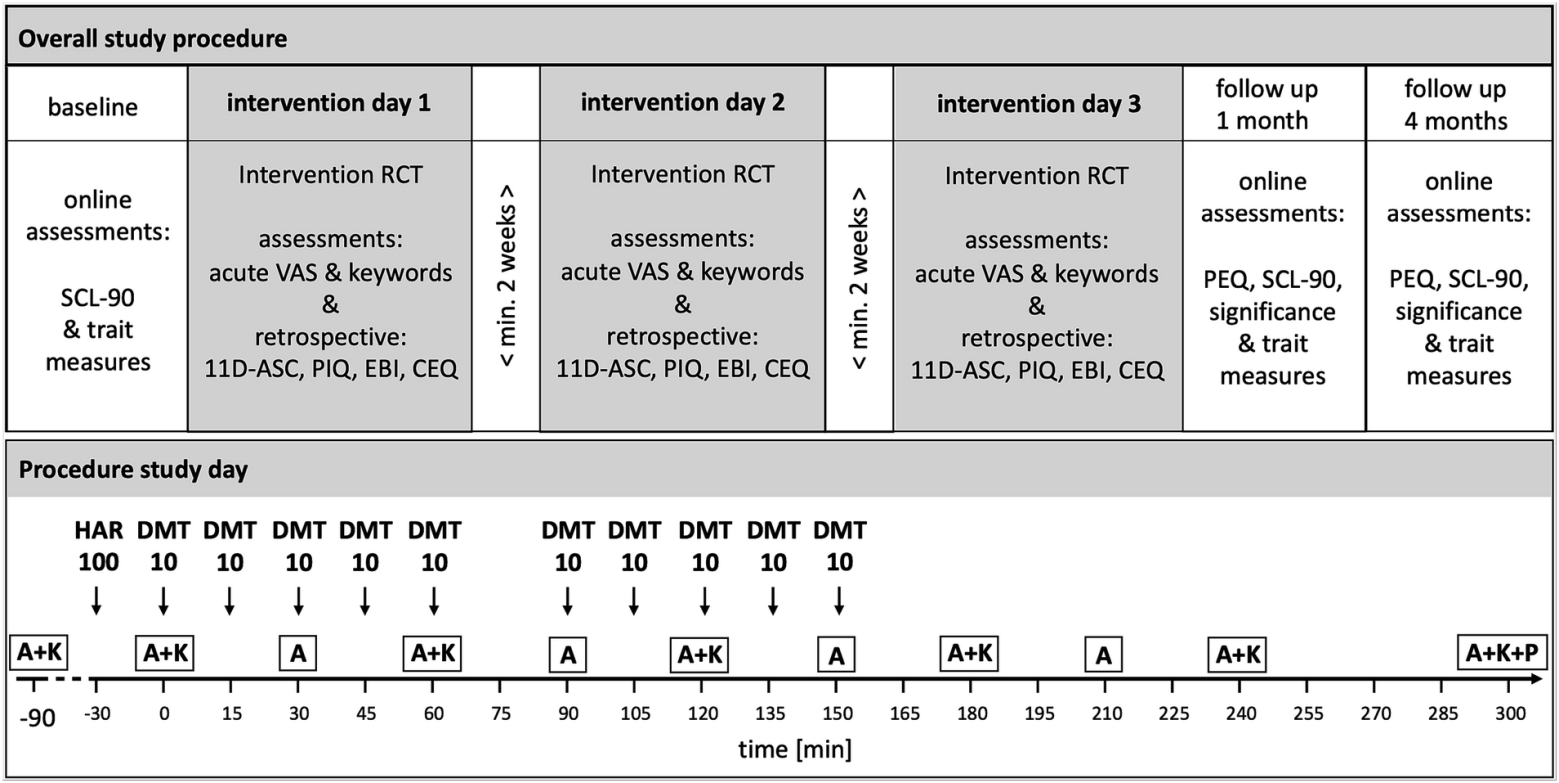
Overview of Study Design, Study Visits and Intervention Days Procedure. HAR = harmine (or placebo respectively); DMT, N = N-dimethyltryptamine (or placebo respectively). Drug conditions were randomized, double-blind, crossover: (1) Harmine 100 mg buccal + DMT 100 mg (10* 10 mg) intranasal; (2) Harmine 100 mg buccal + Placebo intranasal; (3) Placebo buccal + Placebo intranasal. Intranasal: Repeated intermittent dosing. Washout-phase of at least 2 weeks between intervention days. A = acute assessment; K = keywords experience sampling; P = post-acute assessment. A&K experience sampling was assessed with VAS (visual analog scale) items. SCL-90-R: Symptom check list; 5D-ASC = 5 dimensions altered states of consciousness rating scale; PIQ = psychological insights questionnaire; EBI = emotional breakthrough inventory; CEQ = challenging experience questionnaire; PEQ = persisting effects questionnaire. Only assessments relevant for this publication are shown.

The protocol was designed as a randomized, double blind, placebo-controlled crossover study with three conditions performed at three different intervention days with a washout phase of at least 2 weeks between intervention days: (1) DMT + harmine = DMT/HAR, (2) placebo + harmine = HAR, and (3) placebo + placebo = PLA. After informed consent, participants underwent a medical and psychiatric screening. Before the first intervention day, they filled out a baseline online assessment consisting of several questionnaires. At the intervention day, participants arrived at 10 am or 11 am. After arrival, participants filled out questionnaires assessing their current state. Measurement devices for further data collection (electroencephalography, electrocardiography, and venflon for blood sampling) were installed. Dosing of the study drug (harmine or placebo administration) was started at 11:30 am (12,30 am respectively, in the case of parallel testing of two subjects in two separate, but identical rooms). Throughout the day, acute experience sampling was performed. Around t300 (afterglow phase; t referring to *minutes after initial DMT or placebo administration*), post-acute retrospective questionnaires regarding the subjective drug-induced experience were filled out on a tablet. The procedure of the intervention day was identical for all three drug conditions. At 1 month and 4 months after the last intervention day, participants filled out online follow-up questionnaires. An overview of the general study procedure including study visits and schematic procedure of the intervention day is shown in Figure 1.

### 2.3 Setting

The study was conducted during daytime in the Human Sleep Laboratories of the University of Zurich. The soundproof and temperature-controlled rooms were set up in a comfortable living room atmosphere and equipped with dimmable colorful lights and a sound system. Technical monitoring and measuring devices like blood pressure monitor, blood withdrawal materials, and EEG were installed. Throughout all study days, a standardized playlist^1^ containing mostly instrumental background music was played to provide a feeling of comfort and relaxation (music was switched off during tasks), while participants sat in a comfortable position on a mattress leaning against a sturdy pillow. A medically and psychologically trained investigator was present in the room at all times during the intervention day.

### 2.4 Study drug and dose regimen

We applied purified forms of DMT (extracted from the plant *Mimosa hostilis*), and synthesized harmine via parenteral administration routes and delivery mechanisms. The combination DMT plus harmine is commonly called “ayahuasca analog” or “pharmahuasca.” To bypass the GI tract and first-pass metabolism in order to improve absorption kinetics, harmine was administered in orodispersible tablets (ODT) for buccal delivery, whereas DMT was formulated as intranasal spray solution to enable incremental dosing. Detailed information on the study drug, administration, and pharmacokinetics/pharmacodynamics (PK-PD) results are and will be published separately [e.g., (56)]. Thirty minutes after administration of harmine HCl (100 mg) or placebo, respectively (t0), the repeated intermittent dosing of DMT or placebo was initiated with a total of 100 mg DMT in intervals of 15 min (10 mg each timepoint; except at t75 due to performance of a behavioral task) over a period of 150 min. At each dosing timepoint, participants had the possibility to discontinue DMT administration or chose only 5 mg and potentially continue at the next time point for improving overall tolerability. The option to skip a dosing was chosen twice over the full study, the option of taking only 5 mg was never used (consequently, two participants had a cumulative dose of 90 mg DMT instead of 100 mg). Administration timepoints are shown in Figure 1.

### 2.5 Outcome measures

The measures included in this publication are mostly widely used, validated psychometric instruments [for an overview see (57)]. Additionally, we developed experience sampling items based on an online survey describing and differentiating between psychedelic and empathogenic aspects of the psychedelic experience. For questionnaires that were not available in German (PIQ, EBI, CEQ, PEQ), we applied the state-of-the-art translation–back-translation approach with two independent translators for each step to obtain a German version.

#### 2.5.1 Online baseline measures

The Symptom Check-List-90-Revised [SCL-90-R (58, 59);] is a multidimensional psychological screening instrument where subjects rate 90 symptoms of distress and psychopathology on a 5-point Likert-scale with 0 = “not at all” and 4 = “extremely.” The subscales are somatization, obsessive compulsion, interpersonal sensitivity, depression, anxiety, hostility, phobic anxiety, paranoid ideation, and psychoticism.

#### 2.5.2 Acute subjective experience

The overall acute psychedelic state was assessed at regular intervals throughout the day with single word and full sentence acute experience sampling items, which were developed to capture the broader phenomenology of the experience during the dynamic progression over time. Participants rated the acute effect of the drug or placebo along the following items: *any substance-induced effects* (answer format: yes/no), *progression* (*since last assessment timepoint;* answer format: increase, same, decrease), *intensity, liking, disliking, frightening, arousing, relaxing*. Furthermore, they rated four sentences: “I am currently experiencing visual effects (patterns, colors, shapes) induced by the substance with my eyes open and closed” (*elementary imagery*), “I currently experience through the substance sceneries, landscapes, stories in front of my inner eye/in my imagination” (*complex imagery*), “I feel like I’m uncomfortably losing control” (*loss of control*), “I feel like I can comfortably let go/relinquish control” (*letting go*). Additionally, participants rated how easy/clear or how difficult/unclear it was for them to answer these single-word and sentence items (*ambiguity*), which referred to the experience of contradictions within their phenomenal experiences and a difficulty to define a clear value for each item. If not otherwise specified, these items (single words and sentences) were rated on a 0–100 VAS scale on a tablet with touchscreen. Assessments took place before drug administration (day baseline), at t0 (right before the first DMT/placebo administration, which corresponds to 30 min after harmine/placebo administration), and then every 30 min until t240, and a last time at t300, corresponding to afterglow phase.

Complementary, before drug administration (day baseline), at t0, and then every 60 min until t300, participants rated 20 keywords (single word items) referring to the psychedelic and empathogenic phenomenology of the experience on a 0–100 VAS. The selection of items for the two constructs «psychedelic» (*hallucinating, visuals, scary, patterns, multidimensional, dreamlike, colorful, loss of reality, helpless, constricting, adventurous, sensory overload*) and «empathogenic» (*compassionate, open, heart-warming, pleasurable, unity, loving, tolerant, stable, accepting, connected, harmonious*) was based on a 3-step procedure including an online survey which revealed 2 × 10 items with the highest discriminatory power for the two constructs. The development of this psychedelic/empathogenic keywords experience sampling tool will be described in a separate publication. Additionally, at these timepoints, perceived body boundaries were measured with the Perceived Body Boundaries Scale [PBBS; (60)].

#### 2.5.3 Intervention days post-acute retrospective measures

Participants filled out retrospective questionnaires regarding the phenomenology of their experience around t300 (5D-ASC, EBI, CEQ), and 1 day after the experience (PIQ).

Participants’ subjective state of consciousness was assessed using the Altered States of Consciousness questionnaire [5D-ASC, (61, 62)]. Studerus et al. (63) conducted a confirmatory factor analysis and revealed 11 subscales (5D-ASC): Experience of unity, spiritual experience, blissful state, insightfulness, changed meaning of percepts, disembodiment, impaired control and cognition, anxiety, elementary imagery, complex imagery, audio-visual synesthesia. The ASC global score was calculated as the mean of all 5D-ASC items except for the items composing the *vigilance reduction* dimension.

The Psychological Insights Questionnaire [PIQ; (26)] assesses the degree to which acute insights were experienced. It consists of two subscales: Avoidance and maladaptive patterns insights (AMP), and goals and adaptive patterns insights (GAP), and a global score. Participants rated each of the 20 items on a six-point scale (with 0 = no/not at all, 1 = so slight cannot decide, 2 = slight, 3 = moderate, 4 = strong/equivalent in degree to any other strong experience, 5 = extreme/more than any other time in my life). The global score was calculated as the mean value of both subscales.

The Emotional Breakthrough Inventory [EBI; (27)] is a reliable and validated scale to assess the degree to which a psychedelic experience was characterized by an emotional breakthrough, i.e., an overcoming of challenging emotions or memories and thereby an experience of emotional release. Participants answered the six items composing the EBI on a 0–100 VAS (with 0 = “not at all,” and 100 = “very much”).

The challenging experience questionnaire [CEQ; (64, 65)] was developed based on user’s experiences with psilocybin (internet survey) and has demonstrated good internal consistency and external reliability. Its global score consists of 26-items forming seven subscales: Fear, grief, physical distress, insanity, isolation, death, paranoia; and a global score. Participants rated the items regarding the challenging experiences on a 0–5 Likert scale (with 0 = “None, not at all” and 5 = “Extreme, more than ever before in my life”). Scores for each subscale were calculated as the mean of the items of the respective subscale, and results are presented in percentages. The global score was calculated as the mean value of all subscales.

#### 2.5.4 Online follow-up 1 month and 4 months

Approximately 1 and 4 months following the last study intervention day, participants filled out a set of online questionnaires. These included the SCL-90-R (for baseline-follow-up comparison; described above in the *online baseline measures* section), PEQ and GSR.

The Persisting Effects Questionnaire [PEQ; (66)] is used to measure long-lasting positive and negative effects (potential changes) subjectively attributed to the psychedelic experience. Items are rated using a 6-point Likert scale from 0 (“none/not at all”) to 5 (“extreme/more than any other time in my life and more than a rating of 4”). The subscales include positive attitudes about life and/or self (17 items), negative attitudes about life and/or self (17 items), positive mood changes (4 items), negative mood changes (4 items), altruistic/positive social effects (eight items), antisocial/negative social effects (eight items), positive behavior changes (1 item), and negative behavior changes (1 item). Results are calculated as percentage of the maximum possible score.

Additionally, we assessed Griffiths’ and colleagues (2006) three questions regarding the subjective significance of the experience (Griffiths significance ratings; GSR), with the first shown here as an example: (1) “How personally meaningful was the experience?” (1 = “no more than routine, everyday experiences”; 2 = “similar to meaningful experiences that occur on average once or more a week”; 3 = “similar to meaningful experiences that occur on average once a month”; 4 = “similar to meaningful experiences that occur on average once a year”; 5 = “similar to meaningful experiences that occur on average once every 5 years”; 6 = “among the 10 most meaningful experiences of my life”; 7 = “among the 5 most meaningful experiences of my life”; and 8 = “the single most meaningful experience of my life”).

#### 2.5.5 Trait measures

At baseline and at both follow-up (1 and 4 months) timepoints, several trait measures were assessed: acceptance and action [AAQ2; (67)], cognitive flexibility [CFI; (68)], nature relatedness [NR6; (69)], World Health Organization–Five Well-being Index [WHO-5; (70)], and openness and neuroticism [Big 5, measured with 10 items each from the International Personality Item Pool IPIP; (71)]. We hypothesized that all trait measures would increase except for neuroticism, which was expected to decrease.

#### 2.5.6 Qualitative diary reports

In order to complement the quantitative results and increase the tangibility, comprehensiveness, and the contextual understanding, we included some illustrative and representative quotes from the diary entries of our participants in the discussion of results. Further diary reports can be found in the Supplementary material.

### 2.6 Statistical analyses

The data were analyzed and visualized with R Studio version 2021.09.2 + 382 (72). Acute effect over time were visualized based on descriptive statistical parameters. Based on check of assumptions for ANOVAs (normality check, Shapiro–Wilk test) or considering the central limit theorem when analyzing the full dataset for all acute and retrospective measures (*n* = 31), mixed model ANOVAs [Type 3 tests, Satterthwaite’s method [S-method]; calculated with the R-package afex, mixed function; (73)] and post-hoc estimated marginal means [EMMs; emmeans R-package, (74); degrees-of-freedom method: asymptotic; value of *p* adjustment for contrasts calculated with estimated marginal means: Tukey method to correct for comparing a family of 3 estimates] were performed to investigate drug effects. Based on check of assumptions for ANOVAs (normality check, Shapiro wilk test), Wilcoxon tests (with Benjamini-Hochberg correction for multiple comparison) for the comparison of two timepoints (follow up 1 and 4 months) and Friedman tests with post-hoc Dunn-Bonferroni tests (Multiple comparison contrasts with Benjamini-Hochberg correction) for the comparison of three timepoints (baseline, follow up 1 and 4 months) were performed to investigate measurement timepoint effects. Incomplete participants’ datasets were excluded from the analysis (comparison of timepoints), which concerned PEQ and SCL-90-R data. According to a median split of the maximum subjective intensity ratings in the DMT/HAR conditions, participants were grouped into low vs. high sensitivity to the study drug. This was used to investigate potential intensity-response effects on the GSR at follow-up and trait changes from baseline to follow-up. For trait measures also interaction effects of sensitivity (low vs. high) * timepoint were calculated. Pearson or spearman correlations were performed (as appropriate according to Shapiro wilk tests) to analyze associations between acute and persisting effects. The significance level was set to *p* = 0.05.

## 3 Results

### 3.1 Acute experience sampling

**Figure 2:**
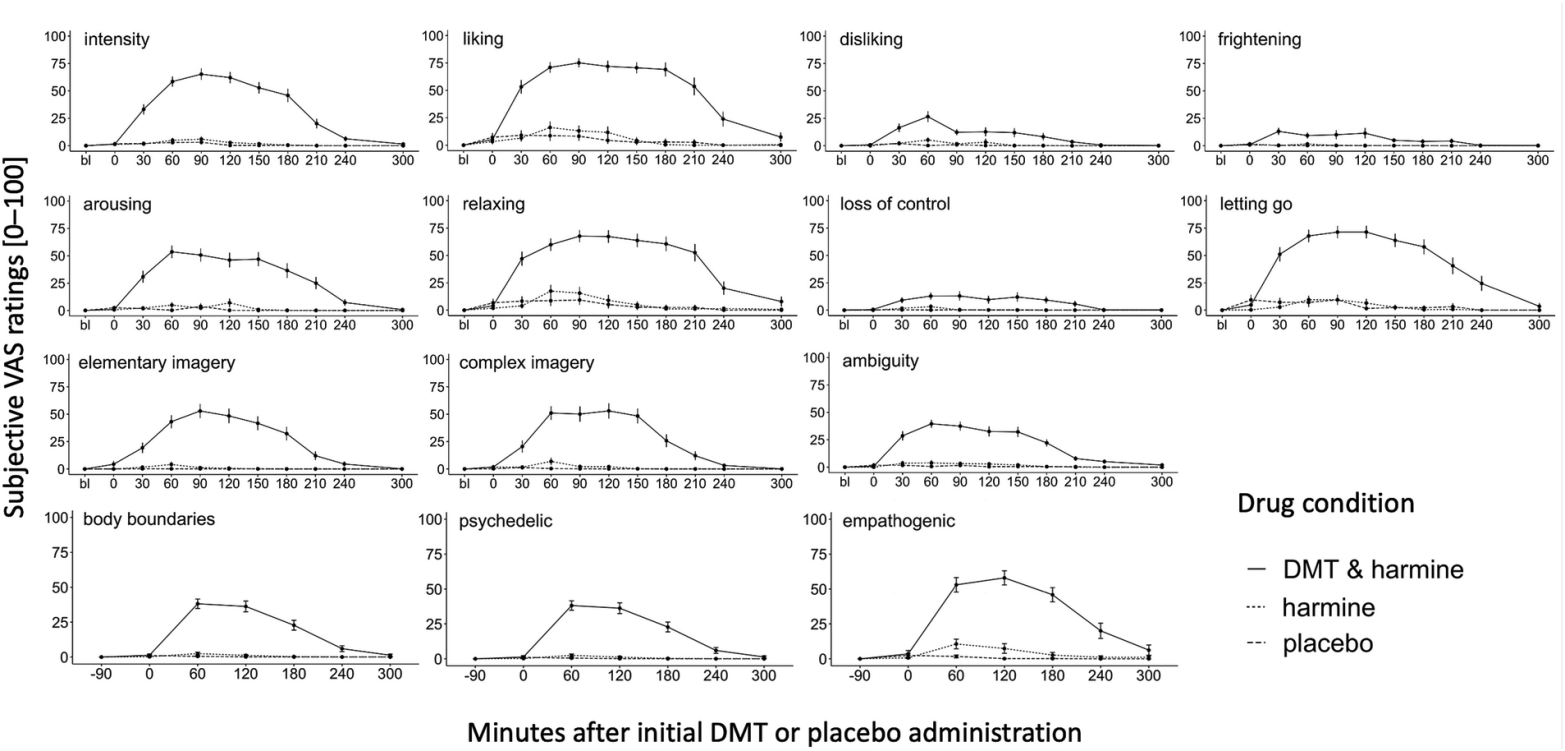
Dynamic Experience Sampling of the Acute Subjective Drug Effects Over Time. Experience sampling: Dynamic ratings of the acute subjective drug effects over time for the 3 conditions DMT/HAR, HAR, PLA. Single word acute items (intensity, liking, disliking, frightening, arousing, relaxing), full sentence acute items (loss of control, letting go, elementary imagery, complex imagery), and ambiguity (how easy/difficult was it to answer the questions) were assessed before drug administration at day baseline (bl), at t0 (right before initial DMT/placebo administration, 30 min after harmine/placebo administration), and every 30 min until t240, and at t300. Psychedelic and empathogenic keywords (ten single words) and body boundaries were assessed at day baseline (bl), t0, and every 60 min until t300. Mean values and standard errors (SEs) are shown.

Figure 2 shows the dynamic evolution of acute drug effects over time for each of the assessed experiential dimensions assessed with the acute experience sampling.

#### 3.1.1 DMT + harmine condition

In the DMT/HAR condition, participants’ subjective peak intensity was reached on average at t90 after the initial DMT administration. Overall, stronger subjective qualities such as *liking, relaxing,* and *letting go* were reported compared to qualities such as *disliking, frightening,* and *loss of control*. Although at a low level, values of disliking peaked t60. Additionally, the subjective experience was rated as arousing and participants reported elementary (patterns, colors, shapes) and complex (landscapes, sceneries, visual memories) imagery and participants reported a sense of body-boundary dissolution. Furthermore, the drug-induced state was experienced as ambiguous. While the overall intensity decreased on average at t90, the positive phenomenal aspects were experienced with a prolonged plateau. Descriptively there were still afterglow residuals of some positive experiential aspects (liking, relaxing, empathogenic), however at t300 differences between DMT/HAR, HAR, and PLA were not significant anymore. Individual progressions of subjective overall drug intensity assessed retrospectively (after t300) can be found in the Supplementary material.

While the *psychedelic* effects peaked on average at t60 and slowly decreased afterwards (stronger decrease after t120, almost back to baseline at t240), the *empathogenic* effects were generally stronger (at peak psychedelic = t60: *t(df)* = 2.12(163), *p* = 0.035; at peak empathogenic = t120: *t(df)* = 2.33(158), *p* = 0.021), increased similarly to the psychedelic effects, remained at a plateau with a peak at t120 and were still reported at t300 (positive afterglow effect).

#### 3.1.2 Harmine only condition

The activation of DMT was achievable with the HAR dose of 100 mg, however this moderate dose of harmine alone–as predicted–did not yield noticable effects. A trend effect of HAR on empathogenic phenomenology at t60 (EMM = 8.94, *p* = 0.062) represented the strongest difference between HAR and PLA. We consider 100 mg of harmine a moderate dose based on results of our dose-finding pilot study (56), where we tested different doses of harmine and demonstrated that doses of 200 mg harmine induced a reduction in vigilance, which was deemed non-conducive to our experimental tasks.

### 3.2 Post-acute retrospective description of the acute subjective drug effects

There was a significant drug effect on all 11 subscales of the 5D-ASC [global score: *F*(2, 60) = 80.21, *p* < 0.001; experience of unity: *F*(2, 60) = 42.14, *p* < 0.001; spiritual experience: *F*(2, 60) = 26.35, *p* < 0.001; blissful state: *F*(2, 60) = 60.48, *p* < 0.001; insightfulness: *F*(2, 60) = 41.28, *p* < 0.001; disembodiment: *F*(2, 60) = 38.84, *p* < 0.001; impaired control and cognition: *F*(2, 60) = 28.52, *p* < 0.001; anxiety: *F*(2, 60) = 9.91, *p* < 0.001; elementary imagery: *F*(2, 60) = 175.98, *p* < 0.001; complex imagery: *F*(2, 60) = 87.32, *p* < 0.001; audio-visual synesthesia: *F*(2, 60) = 38.42, *p* < 0.001; changed meaning of percepts: *F*(2, 60) = 45.43, *p* < 0.001]. Post-hoc tests revealed all subscales of the 5D-ASC were significantly increased by DMT/HAR compared to HAR (global score: *EMM* = 25.55, *p* < 0.001) and compared to PLA (global score: *EMM* = 26.29, *p* < 0.001). No differences were reported between HAR and PLA (global score: *EMM* = 0.74, *p* = 0.95).

There was a significant drug effect on the PIQ global score [*F*(2, 58.5) = 28.51, *p* < 0.001] and both subscales AMP [*F*(2, 58.5) = 22.49, *p* < 0.001] and GAP [*F*(2, 58.5) = 32.59, *p* < 0.001]. Post-hoc tests revealed the global score and both subscales of the PIQ were significantly increased by DMT/HAR compared to HAR (global score: *EMM* = 1.12, *p* < 0.001) and compared to PLA (global score: *EMM* = 1.28, *p* < 0.001). No differences were reported between HAR and PLA (global score: *EMM* = 0.16, *p* = 0.69).

There was a significant drug effect on the EBI [*F*(2, 60) = 26.51, *p* < 0.001]. Post-hoc tests revealed a significant increase of the EBI by DMT/HAR compared to HAR (*EMM* = 24.49, *p* < 0.001) and compared to PLA (*EMM* = 25.78, *p* < 0.001). No differences were reported between HAR and PLA (*EMM* = 1.28, *p* = 0.94).

There was a significant drug effect on the CEQ global score [*F*(2, 60) = 12.84, *p* < 0.001] and most subscales [fear: *F*(2, 60) = 5.09, *p* = 0.009; grief: *F*(2, 60) = 4.28, *p* = 0.018; physical distress: *F*(2, 60) = 14.01, *p* < 0.001; insanity: *F*(2, 60) = 7.86, *p* < 0.001; death: *F*(2, 60) = 3.94, *p* = 0.023], but not for the subscales insolation [*F*(2, 60) = 1.86, *p* = 0.165] and paranoia [*F*(2, 60) = 1.97, *p* = 0.15]. Post-hoc tests revealed a significant increase of the CEQ by DMT/HAR compared to HAR (*EMM* = 4.48, *p* < 0.001) and compared to PLA (EMM = 5.13, *p* < 0.001). No differences were reported between HAR and PLA (EMM = 0.65, *p* = 0.83).

**Figure 3:**
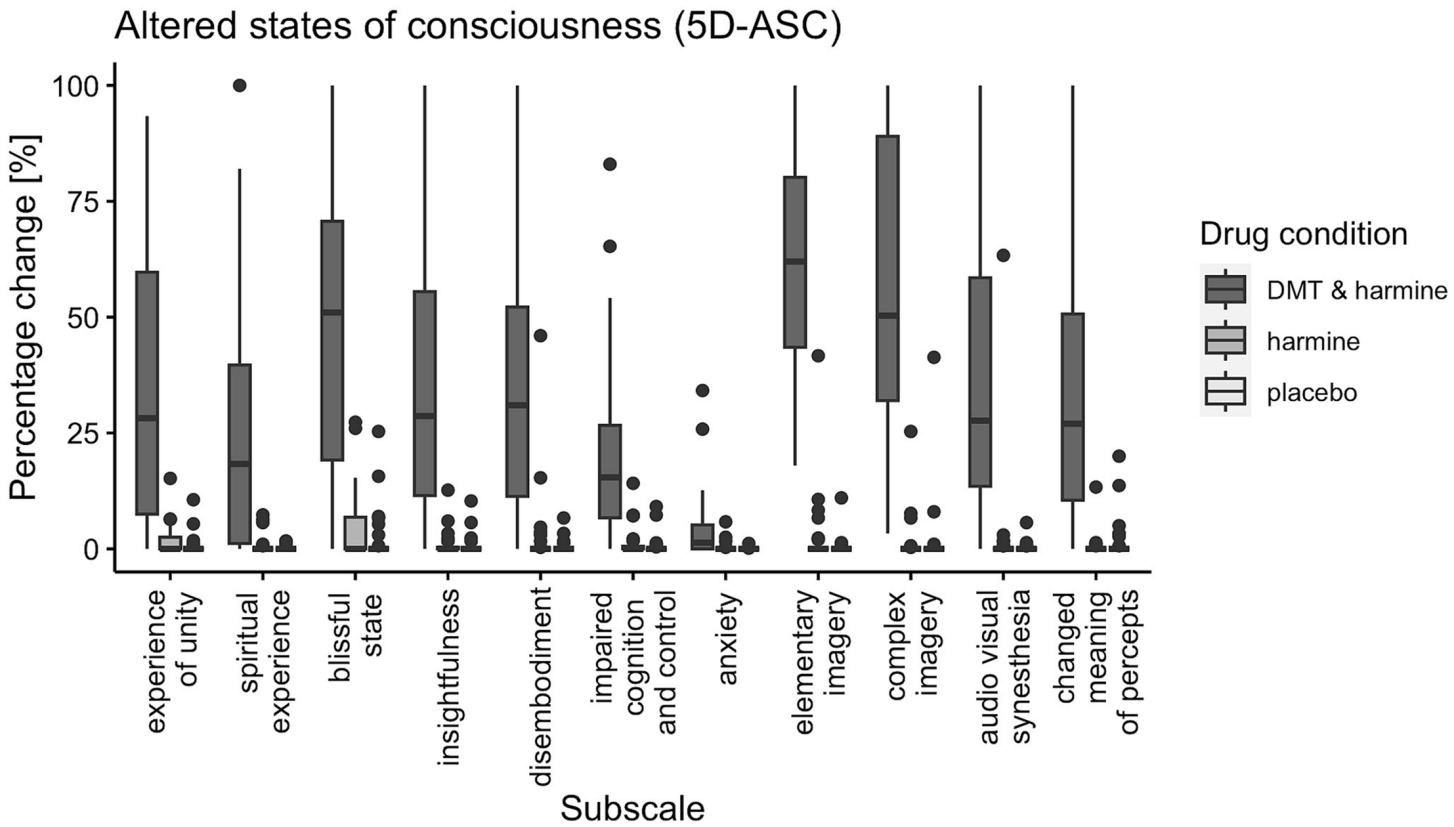
Participant’s Retrospective Ratings on the 5D-ASC Questionnaire. 5-Dimensions Altered States of Consciousness rating scale (5D-ASC) for all three drug conditions (DMT/HAR, HAR, PLA) with boxplots for all subscales: experience of unity, spiritual experience, blissful state, insightfulness, disembodiment, impaired cognition and control, anxiety, elementary imagery, complex imagery, audio-visual synesthesia, changed meaning of percepts. Spider plot visualizations of the 11 subscales and the 5 dimensions of the 5D-ASC for comparability with other trials using the same scale can be found in the Supplementary Figure S1.

**Figure 4:**
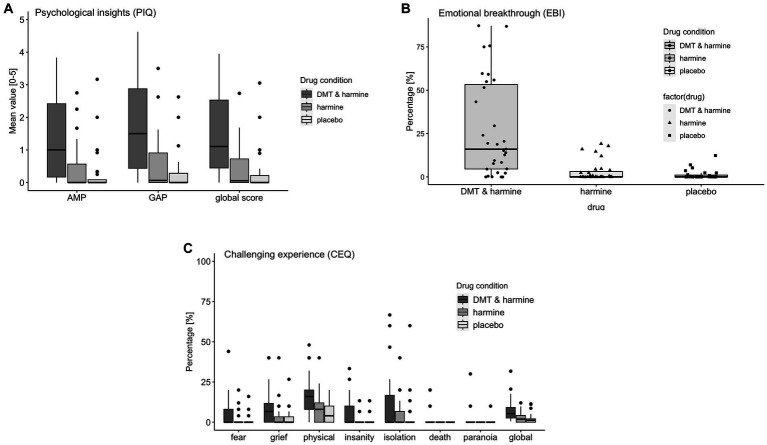
Retrospective Assessment of the Subjective Experience. Retrospective assessment of the subjective experience induced by the three drug conditions DMT/HAR = DMT + harmine, HAR = harmine only, PLA = placebo. **(A)** PIQ = psychological insights questionnaire; AMP = avoidance and maladaptive patterns insights; GAP = goals and adaptive patterns insights, global score; **(B)** EBI = emotional breakthrough inventory; **(C)** CEQ = challenging experience questionnaire fear, grief, physical distress, insanity, isolation, death, paranoia, global score.

Results are visualized in Figure 3 (ASC) and Figures 4A–C (PIQ, EBI, CEQ). A table of results for all scales, subscales, and contrasts can be found in the Supplementary Table S5.

### 3.3 Persisting effects

**Figure 5:**
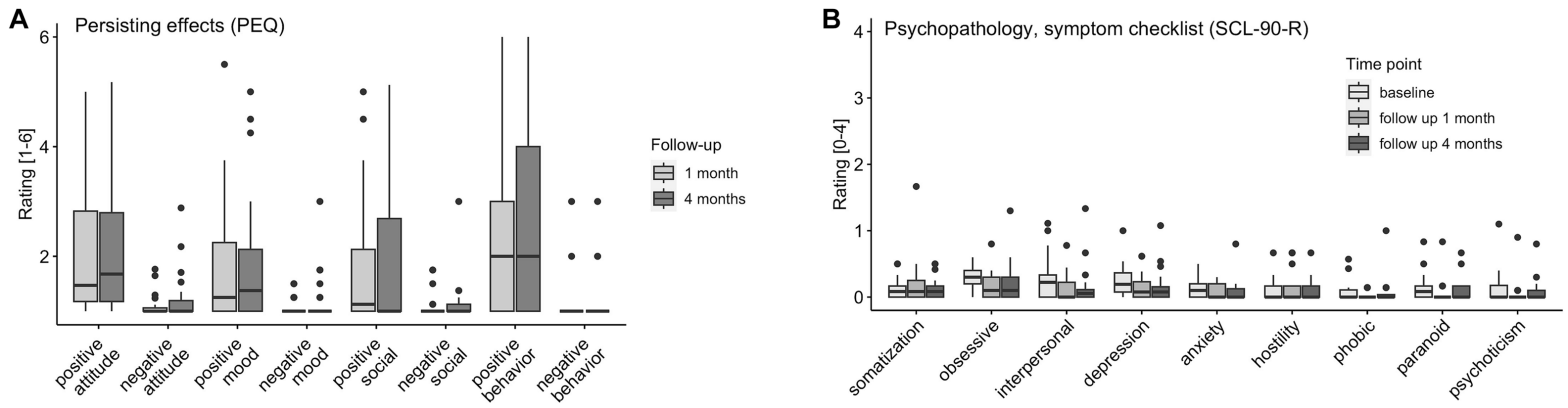
Persisting Effects and Symptoms. **(A)** PEQ (persisting effects) assessed at 1 month (*n* = 26) and 4 months (*n* = 28) follow-up. **(B)** SCL-90-R (symptom checklist) assessed at baseline (*n* = 31), 1 month (*n* = 25), and 4 months (*n* = 28) follow-up: somatization, obsessive compulsion, interpersonal sensitivity, depression, anxiety, hostility, phobic anxiety, paranoid ideation, psychoticism.

Participants reported significantly stronger positive compared to negative persisting effects for all four domains (both follow-up timepoints combined attitude toward life: *r* = 0.83, *p* < 0.001; mood: *r* = 0.57, *p* < 0.001, social effects: *r* = 0.46, *p* < 0.001, behavior change: *r* = 0.78, *p* < 0.001) of the PEQ at the follow-up timepoints 1 (*n* = 26) and 4 months (*n* = 28) after the last study intervention day. Results are visualized in Figure 5A and reported in the Supplementary Table S6.

There were main effects of timepoint on the SCL-90-R subscales obsessive compulsion (*χ^2^*(2) = 9.18, *p* = 0.01), interpersonal sensitivity [*χ^2^*(2) = 11.41, *p* = 0.003], anxiety [*χ^2^*(2) = 6.78, *p* = 0.034], and psychoticism [*χ^2^*(2) = 6.65, *p* = 0.036]. No increases from baseline to follow-up were reported. Overall (for the SCL-90-R subscales obsessive compulsion and paranoid ideation subscales; and interpersonal, depression, and psychoticism subscales, but not after correction for multiple comparison), symptoms significantly decreased from baseline to follow-up 1 month (*n* = 25) and stayed at the lower level [no differences between follow-up 1 month to 4 months (*n* = 28)]. Results are visualized in Figure 5B and reported extensively in the Supplementary Table S6.

**Figure 6:**
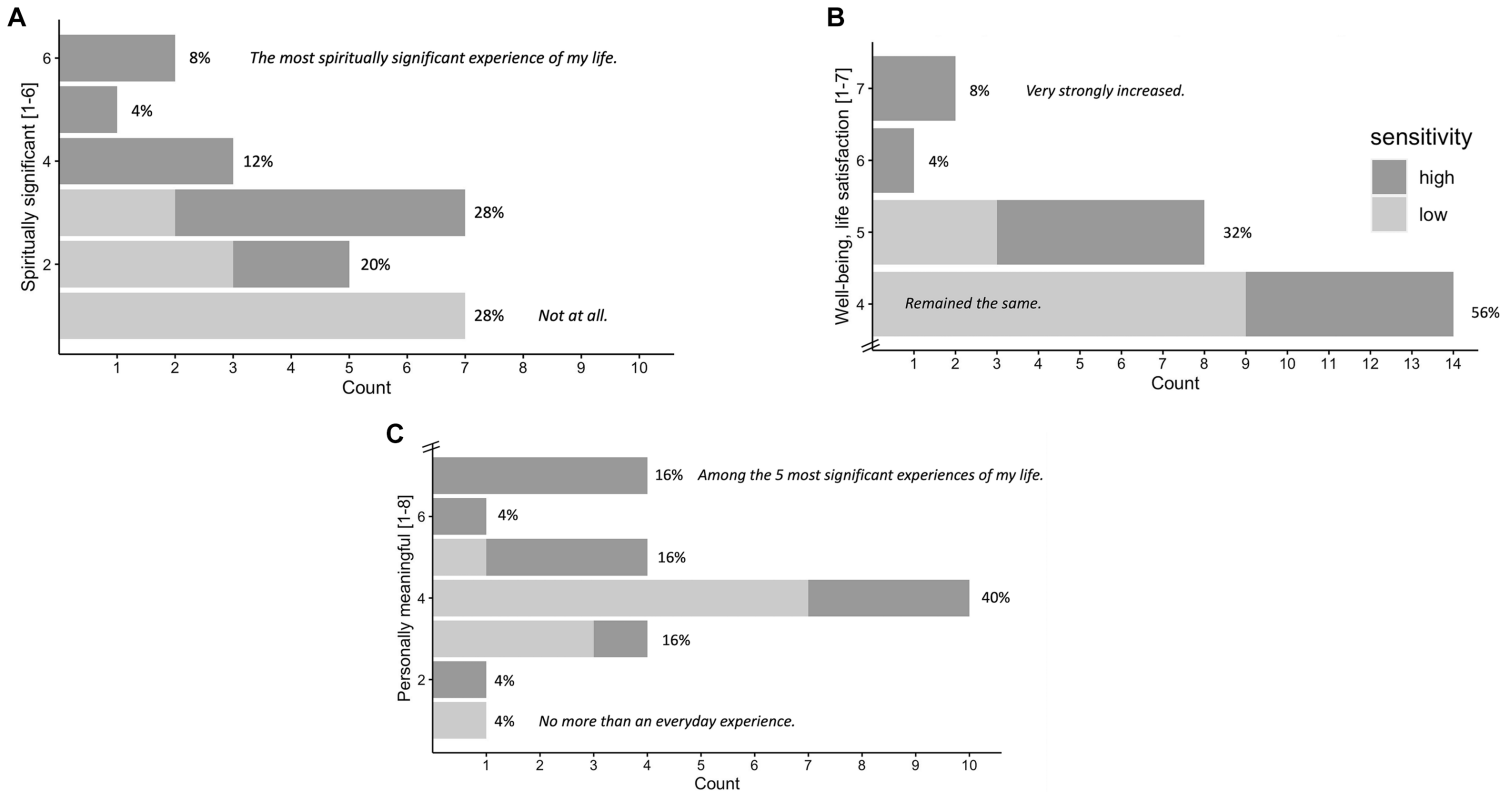
Significance of the Experience Rated at 1 Month Follow-up. *N* = 25. Attribution of subjective significance (GSR) of the experience at 1 month follow-up: Personal meaning **(A)**, spiritual significance **(B)**, change in sense of personal well-being and life satisfaction **(C)**, shown in number and percentage of participants. High vs. low sensitivity group (median split according to maximum subjective intensity ratings) are marked in gray tones, participants of the high sensitivity group generally reporting higher significance. Items and answer options were as follows: **(A)** “How personally meaningful was the experience?” (1 = no more than routine, everyday experiences, 2 = similar to meaningful experiences that occur on average once or more a week; 3 = similar to meaningful experiences that occur on average once a month; 4 = similar to meaningful experiences that occur on average once a year; 5 = similar to meaningful experiences that occur on average once every 5 years; 6 = among the 10 most meaningful experiences of my life; 7 = among the 5 most meaningful experiences of my life; and 8 = the single most meaningful experience of my life). **(B)** “Indicate the degree to which the experience was spiritually significant to you.” (1 = not at all, 2 = slightly, 3 = moderately, 4 = very much, 5 = among the 5 most spiritually significant experiences of my life, and 6 = the single most spiritually significant experience of my life). **(C)** “Do you believe that the experience and your contemplation of that experience have led to change in your current sense of personal well-being or life satisfaction?” (7 = increased very much,6 = increased moderately, 5 = increased slightly, 4 = no change, 3 = decreased slightly, 2 = decreased moderately, and 1 = decreased very much). GSR at 4 months follow-up can be found in the Supplementary Figure S2.

Results of the GSR (frequency counts) at 1 month follow-up are visualized in Figures 6A–C. *N* = 25 participants filled out 1 month follow-up GSR. Results for the GSR 4 months follow-up can be found in the Supplementary Figure S2.

### 3.4 Trait measure changes from baseline to follow-up

*N* = 22 participants filled out all follow-up trait assessments. We did not find increases from baseline to follow-up 1 month and 4 months for the trait measures acceptance and action (AAQ2), cognitive flexibility (CFI), nature relatedness (NR6), and well-being (WHO-5), nor openness (IPIP), and no decreases in neuroticism (IPIP), for the full sample nor for the two subgroups of high vs. low sensitivity, nor for the drug-naïve subsample. Trait measures at baseline and both follow-up timepoints 1 and 4 months are visualized in the Supplementary Figure S3.

### 3.5 Correlations

We found positive correlations of most psychometric retrospective measures of the acute experience (ASC, PIQ, EBI) and persisting effects (PEQ) and significance ratings (GSR). However, CEQ did not correlate significantly with any of the other measures, potentially because of the low values of CEQ. The results are shown in Table 1. Furthermore, within the DMT/HAR condition, we found a significant positive correlation between *progression* with the experience sampling items *disliking* (*rho* = 0.664, *p* < 0.001), *frightening* (*rho* = 0.577, *p* < 0.001), and *loss of control* (*rho* = 0.633, *p* < 0.001).

**Table 1:** Correlations of subjective experience with persisting effects at 1 month follow-up.

		Persisting effects				
		PEQ positive	PEQ negative	Significance: meaning	Significance: spiritual	Significance: well-being
Subjective experience	ASC	0.725***	0.268	0.712***	0.583**	0.635***
	PIQ	0.788***	0.341	0.529**	0.582**	0.746***
	AMP	0.739***	0.395	0.488*	0.482*	0.662***
	GAP	0.762***	0.204	0.504*	0.647***	0.776***
	EBI	0.691***	0.381	0.471*	0.521**	0.735***
	CEQ	0.431*	0.286	0.361	0.469*	0.399*

*p < 0.1, **p < 0.05, ***p < 0.01. ASC, global score altered states of consciousness rating scale; PIQ, psychological insights questionnaire. AMP, avoidance and maladaptive patterns; GAP, goals and adaptive patterns; EBI, emotional breakthrough inventory; CEQ, challenging experience questionnaire; PEQ, persisting effects positive and negative; significance: personally meaningful, spiritually significant, contribution to well-being. Pearson correlation coefficients.

## 4 Discussion

This is the first randomized controlled trial investigating the subjective experience profile and psychological change processes of an ayahuasca-inspired formulation containing DMT and harmine in healthy participants. Specifically, we compared the effects of DMT + harmine (DMT/HAR), harmine only (HAR), and placebo (PLA) by sampling the acute subjective experience and assessing alterations of consciousness (5D-ASC). Our main goal was to evaluate surrogate markers of potential psychotherapeutic efficacy and psychological change mechanisms using psychometric tools such as the psychological insights questionnaire (PIQ), the emotional breakthrough inventory (EBI), psychological safety and tolerability (CEQ, SCL-90-R), persisting effects (PEQ), and attributed significance. Importantly, DMT (combined with harmine) was the main driver for the assessed effects, while harmine alone led to only small or mostly negligible effects.

### 4.1 Subjective experience and dynamic progression of subjective effects

Overall, the subjective experience shared phenomenological similarities with ayahuasca and other serotonergic psychedelics such as psilocybin (19, 49), including alterations of consciousness captured by the 11 subscales of the widely used 5D-ASC scale (63, 75). Experience sampling over the course of acute drug effects revealed experiences characterized by attributes such as liking, letting go, relaxation, and visionary experiences (elementary and complex imagery). Some levels of arousal and only low levels of disliking, frightening, and loss of control were reported. The average peak of disliking was experienced at the timepoint of a task performed short before the average peak of the drug effect, which might have influenced the experience.

Our findings suggest that the incremental intermittent dosing regimen might have affected the subjective experience profile. The clear biphasic phenomenology of initially psychedelic and potentially overwhelming, and subsequently more empathogenic experiences, described for ayahuasca such as discussed by Wolff (19, 49) as *“*bottleneck*”* phenomenon during the ayahuasca experience was less prominent in our study. The bottleneck phenomenon refers to sudden transformations in the quality and intensity of the ayahuasca-induced experience after vomiting (49). Due to the rare occurrence of vomiting (1 participant) the biphasic phenomenology might be attenuated with our DMT/HAR formulation and administration protocol. Mostly moderate psychedelic effects (assessed with experience sampling) were reported in the early phases of the experience. Empathogenic qualities (assessed with experience sampling) were reported more prominently compared to psychedelic qualities during the entire session and to some degree also during the afterglow phase (at t300, when intensity levels were back to baseline). Based on our findings, we argue that our administration route and incremental dosing regimen of DMT/HAR facilitates an adaptive process, which enables the organism to adjust its psychological homeostasis, which refers to the organisms’ capability to self-regulate when challenged with altering environmental and biochemical conditions, potentially leading to an acute psychological tolerance resulting in a less overwhelming and more empathogenic experience, compared to high bolus administrations.

### 4.2 Therapy-relevant psychological processes

Of great relevance for a potential therapeutic application, DMT/HAR led to psychological insights [PIQ; (26)] and emotional breakthrough [EBI; (27)]. This combination represents a potential for transformative psychological processes and has been discussed to be essential for the improvement of mental health (28, 29). DMT/HAR formulations could thus play a key role in psychedelic-assisted therapy by eliciting both psychological insights and emotional breakthrough experiences.

The qualitative diary reports revealed further insights into beneficial and therapy-relevant effects of the intervention. Recurring topics were related to being present in the “here and now,” clarity, trust, stillness, relaxation, contentment, non-judgment, awareness, universality, acceptance, gratitude, appreciation, and feelings of connectedness. Diary reports can be found in the Supplementary materials and in the following illustrative example:

“I notice how I try to look at emotions and thoughts from a greater distance without judging them. It is difficult, but since the trip one of my goals. I also try to build a greater acceptance for thoughts and decisions and to go through life with what is and not what is not. During the trip I had the strongest feeling ever that everything is ok the way it is.”

### 4.3 Psychological safety and tolerability

The mechanism of homeostasis adjustment due to the incremental dosing mentioned above–i.e., the psychological adaptation to the increasing cumulative DMT dose–might reduce potential psychological adverse effects and allow for better controllability by the participant. We found that the escalation of acute DMT/HAR drug effects (progression change as assessed with experience sampling) was positively correlated with the sensation of fear (frightening) and loss of control, supporting the hypothesis that not only the overall intensity, but also the dynamic change of intensity could potentially be experienced as challenging. With the intranasal DMT application form and the intermittent dosing regimen, the possibly overwhelming effects might have been attenuated and better controllable compared to high bolus administrations. One participant described this in his diary report:

“When I closed my eyes, I could dive into worlds and be really absorbed by that, but as soon as I opened my eyes I could focus on the now again. Talking and interacting with people went very well…”

This was also reflected in the (very) low levels of the *anxiety* and *impaired control and cognition* subscales of the 5D-ASC. While traditional ayahuasca is relatively safe, especially when consumed in a ritualized setting (46), its effects can be unpredictable due to varying combinations and concentrations of plant-based ingredients and interindividual differences in gastrointestinal absorption. In a global ayahuasca survey, 42% of participants reported–mostly transient – challenging emotional-cognitive adverse effects (48) such as feeling disconnected or alone (21%). In our study setting, however, DMT/HAR stimulated only small levels of feelings of loss of control (experience sampling), and only small levels of acute transient challenging experiences (CEQ) and anxiety (5D-ASC). No difference between harmine and placebo were reported. Compared to other psychedelics such as psilocybin (Holze: 5.6 ± 2.2, 6.5 ± 2.4; Ley: 4.9 ± 1.7; Passie 3–6 h) or LSD (Holze: 9.3 ± 3.2, 11 ± 3.7; Ley: 8.2 ± 3.1; Passie: 9–12 h) (39, 76–78), the overall experience duration of combined DMT/HAR with this specific administration protocol (3–4 h) is shorter. In light of the cost-intensive nature of the therapeutic guidance, shorter durations might pose an advantage of innovative DMT formulations over classical psychedelics for clinical applications. Moreover, if repeated-intermittent DMT administration is discontinued, the intensity of the experience subsides rapidly as assessed with the experience sampling items, and therefore the duration is much more flexible, which enhances the controllability and psychological safety profile of the psychedelic intervention. Physiological safety and tolerability data including undesired side effects, vital signs, and PK-PD-profiles will be published separately.

Overall, the combined parenteral DMT/HAR formulation shows a favorable psychological safety profile, which is an important aspect of any therapeutic intervention especially in vulnerable patient populations. These properties, including the option for flexible dosing, make the DMT/HAR formulation an interesting candidate for individualized applications in the framework of precision medicine. Physiological safety and tolerability data will complement the picture, before the DMT/HAR formulation can be tested in patient populations. Drug interactions will have to be considered, especially regarding the MAO-I. Even though observational data suggest that the risk of combining short-acting, reversible MAO-I such as in ayahuasca with antidepressants might be overestimated (79), more research is needed to fully understand the interactions and potential associated risks.

### 4.4 Persisting effects, integration, and contextual factors

Notably, persisting effects reported by participants at the 1- and 4-months follow-up assessment were mainly positive on all subscales: Positive attitudes about life and/or self, positive mood changes, altruistic/positive social effects, and positive behavior changes (PEQ), with the strongest effects for behavioral changes. Only very small levels of negative persisting effects (PEQ, all 4 subscales) were reported in some participants, and they did not require medical attention. Overall, participants attributed personal and spiritual significance and perceived association with increased well-being to their study participation. These primarily positive persistent effects further underline the potential of ayahuasca-inspired formulations to promote transformative experiences with sustained beneficial outcomes on attitudes and behaviors even weeks or months after the intervention. Furthermore, psychopathological symptoms (SCL-90-R) in everyday life were stable or even decreased after the study participation, which underscores that DMT/HAR at the dose administered to healthy participants has no negative long-term consequences on physical or mental health.

However, while other studies found lasting positive changes in personality traits such as openness, neuroticism, cognitive and psychological flexibility, or nature relatedness (80–82), or lasting positive changes in mood and attitude (83) after use of psychedelics, we did not find trait-related improvements in our study sample, despite the reported positive persisting effects. Considering the moderate dose and the neurobehavioral study setting, which do not facilitate an introspective experience, the positive persisting effects (PEQ) are still remarkable. Although the study setting was comfortable, the study did not have a therapy-oriented framework. Emphasizing integration might have a positive impact on the sustainability of any potential positive effects brought about by a psychedelic experience. Integration refers to the process of making sense of and contextualize the insights and emotions that arise during a psychedelic experience into one’s daily life (84). Apart from qualitative interviews after the session, our study protocol did not include any formal integration sessions, however some participants reflected on how to integrate the experience within their life using diary reports. This may have initiated a potentially beneficial process supporting lasting persisting effects, as exemplified by one participant in his diary report:

"The rest of the evening I felt very thoughtful, but in a positive sense. There was just a lot to process and think about again. On the one hand what I experienced, but also how I perceived it and what I can now do with it in everyday life.”

Furthermore, and contrary to the notion of one single dose leading to sustainable changes (30), it might be valuable to offer several psychedelic sessions to deepen insights and processes, as one of our participants’ reports states:

“… difficult to implement everything learned and not to fall back into the same patterns. I have the feeling that you need several such trips and states to be able to really change that sustainably.”

Contextual factors, such as the environment in which the experience takes place (setting), the mindset of the individual (set), and the support available after the experience (integration and setting), have a huge impact on the effects of psychedelics (85–88). Due to the potential of psychedelics to elicit profound psychological and emotional responses, providing empathetic support during and after a psychedelic experience represents a fundamental ethical requirement (84, 89). The diary reports of our participants indicate that they felt safe and supported during the study, which might have contributed to the positive acute and persisting effects and might have prevented negative reactions. Even though empathetic support was provided during the duration of the study, the setting was designed to meet the requirements for neurobehavioral and clinical pharmacology assessments.

### 4.5 Limitations

To our knowledge, this is the first RCT investigating the acute subjective experience profile, psychological change mechanisms and persisting effects of a DMT/HAR formulation and harmine alone in healthy individuals. However, the study has some considerable limitations:

In the present study, we co-administered intranasal DMT with buccal harmine and harmine only; however, we did not assess the effects of intranasal DMT only. As a result, the specific pharmacological outcomes of intranasal DMT remain to be elucidated. It is noteworthy to mention that Turner and Merlis conducted a study in the 1950s wherein they found intranasal DMT to be pharmacologically inactive (54). Based on these findings, it can be postulated that the sole intranasal administration of DMT would likely demonstrate diminished bioavailability, leading to minimal systemic activity. However, in a follow-up study, we included a DMT only condition to address this limitation (manuscripts in preparation).

Due to the nature of psychedelic-induced effects, the effectiveness of blinding might be diminished in our study as well. Expectations might have increased effects of the DMT/HAR condition (90). Furthermore, our sample was Western, educated, industrialized, rich, democratic [WEIRD; (91)] and in our case even male because of the repeated-measures design and potential influence of the female cycle on endocrinological assessments, which limits the generalizability of the results. While other studies investigating psychedelics often include participants with previous psychedelic experience (17, 18), we excluded participants more than 15 lifetime psychedelic experiences resulting in a sample with no or only little previous psychedelic experience. However, all our participants had previously consumed THC, which might also be specific for that specific sample of healthy, 20–40 years old, male participants. A comprehensive overview of participants’ previous drug experience can be found in the Supplementary Tables S3, S4. Another potential selection bias remains, as studies including treatments with psychedelic substances require a rather high degree of motivation and openness to experience (82) by the participants because of the time-demanding study procedure and the phenomenologically rich nature of psychedelic experiences. This might have contributed to a ceiling effect regarding potential changes in trait measures. Less carefully screened participants, outside of a study setting, might experience different acute and persisting effects. Additionally, any potential long-term assessments might have been confounded by the impact that the COVID-19 pandemic and lockdowns had on people’s daily routines, including isolation and decreased social interactions, and mental health (92), or by other life events such as exam periods of our student participants. It is important to notice that only 22 out of 31 participants filled out all follow-up assessments (trait measures), which further limits any interpretation regarding persisting effects or trait-changes.

The pharmacologically and neurobehaviorally oriented study set up was not targeted for transformative experiences, therapeutic or integration processes, but rather required participants to follow frequent study procedures including psychometric assessments, blood withdrawals, and performance of behavioral tasks. For the experience sampling and the completion of the tasks, participants had to open their eyes and to engage with electronic devices, which shifts the focus away from introspection toward a task-related attention, thereby potentially reducing the subjective intensity of the experience. While the intensity of the experience was rather moderate–as expected with the incremental dosing of 10 single doses à 10 mg of DMT [compare also bolus and total doses of other studies, e.g., (18)]–the study set up, including the experimental tasks, might have even attenuated the subjective effects and distracted participants from their inner processes, as reported in some of the diary texts (examples can be found in the Supplementary material).

It will be important to carefully consider both integration and contextual factors when testing DMT/HAR for therapeutic purposes. Future studies, especially clinical trials with patients, should provide a setting that is targeted for psychological processes and integration, potentially even in group settings (93). Such an environment would ideally support individuals to reflect on their experiences, process their emotions and challenging aspects of the experience, and incorporate their insights into their daily lives. This could minimize any psychological adverse reaction and enhance the positive effects we found in our pharmacologically oriented study setting. Further clinical trials in a therapeutic context including preparation and integration and with individualized doses are necessary to evaluate if the effects of ayahuasca-inspired formulations can be enhanced by contextual variables and to assess the clinical potential of DMT/HAR formulations to patient populations.

### 4.6 Conclusion

Taken together, our findings suggest that this novel ayahuasca-inspired formulation containing DMT and harmine, but not harmine only, induce an experience with psychedelic and empathogenic characteristics. Similar to other psychedelics, combined DMT and harmine has the potential to elicit psychological insights (PIQ) and emotional breakthroughs (EBI), with positive persisting effects in healthy subjects. Hence, the DMT/HAR formulation impacts fundamental psychological processes, suggesting a transdiagnostic mechanisms of action which could explain a therapeutic potential beyond specific psychiatric diagnoses across various mental health conditions. Furthermore, we observed a favorable psychological safety and tolerability profile in a supportive setting, marked by attenuation of potentially challenging effects, as well as the unpredictability and interindividual variability of acute drug effects commonly associated with traditional ayahuasca. The phenomenological diversity of psychedelic-induced experiences, the high context-sensitivity and complexity of these states, their underlying mechanisms, and their potentially therapeutic effects raise further interesting research questions for future studies.

## Data availability statement

The datasets presented in this study can be found in online repositories. The names of the repository/repositories and accession number(s) can be found: Open Science Framework: https://osf.io/fvpdn/?view_only=7257b4001b584fe9ac75535a23f6639f.

## Ethics statement

The studies involving humans were approved by Cantonal Ethics Committee of the Canton of Zurich (Basec-Nr. 2018–01385) and Swiss Federal Office of Public Health [BAG-Nr. (AB)-8/5-BetmG-2019/008014]. The studies were conducted in accordance with the local legislation and institutional requirements. The participants provided their written informed consent to participate in this study.

## Author contributions

HA: Conceptualization, Formal analysis, Funding acquisition, Investigation, Methodology, Project administration, Visualization, Writing – original draft, Writing – review & editing, Data curation. MM: Conceptualization, Formal analysis, Funding acquisition, Investigation, Methodology, Project administration, Visualization, Writing – original draft, Writing – review & editing, Data curation. DD: Conceptualization, Funding acquisition, Methodology, Project administration, Supervision, Writing – review & editing. DS: Investigation, Project administration, Writing – review & editing, Data curation. CE: Data curation, Writing – review & editing. IW: Data curation, Writing – review & editing. DM: Data curation, Writing – review & editing. LC: Data curation, Writing – review & editing. AH: Data curation, Writing – review & editing. CS: Data curation, Writing – review & editing. JM: Writing – review & editing, Data curation. RR: Writing – review & editing, Conceptualization, Methodology. BK: Supervision, Validation, Writing – review & editing. MS: Conceptualization, Funding acquisition, Methodology, Project administration, Supervision, Validation, Writing – review & editing.

## Footnotes

^1^ https://tinyurl.com/2p83pkze
