# Supplementary material for: Potential therapeutic effects of an ayahuasca-inspired N,N-DMT and harmine formulation: a controlled trial in healthy subjects
Source: Front Psychiatry. 2024 Jan 8;14:1302559. doi: 10.3389/fpsyt.2023.1302559 (PMC10804806; doi:10.3389/fpsyt.2023.1302559)
Supplement: Supplementary file 1 [file Data_Sheet_1.PDF]

frontiers

Supplementary Material

The Therapeutic Potential of an Ayahuasca Analogue Containing N,N-DMT and Harmine: A Controlled Trial in Healthy Subjects

***†Aicher, H.D.[1,2,3], †Mueller, M.J.[1,3,4], Dornbierer, D.A.[1,5,6], Suay, D.[1,7], Elsner, C[1]., Wicki, I.A.[1], Meling, D.[1,8], Caflisch, L.[1], Hempe, A.[1,9], Steinhart, C.[1], Mueller, J.[1], Von Rotz, R.[1], Kleim, B.[2,10] & Scheidegger, M.[1,3]**

(†Equal contribution and first authorship)

*** Correspondence:** Helena D. Aicher: helena.aicher@uzh.ch

Supplementary Table 1

Sample description: Demographics

		Participants	Dropouts
Number	n	31	6<br>(data from 5)
Gender	male sample		
Mother tongue	(Swiss) German speaking sample; in some cases an additional mother tongue		
Age (years)	*M (SD)*	25.39 (4.21)	27.4 (5.73)
	min, max, NA's	20, 37	22, 35
Sexual orientation	clearly heterosexual	15/25 (60%)	NA
	rather heterosexual	8/25 (32%)	
	bi-sexual	1/25 (4%)	
	rather homosexual	0	
	clearly homosexual	1/25 (4%)	
BMI	*M (SD)*	22.98 (1.89)	22.77 (1.51)
	min, max	19.59, 26.23, 2 NAs	20.9, 24.49
Physical fitness	very fit	9 (29%)	2 (40%)
	fit	17 (54.8%)	3 (60%)
	average	5 (16.1%)	
	no other fitness level ticked		
	sports on a regular basis	30 (96.8%)	5 (100%)
Education (years)	*M (SD)*	13.6 (6.08)	16.6 (4.16)
	min, max	1.5, 23	11, 21
Education (level)	Certificate of Secondary Education	2 (6.5%)	
	Higher School Certificate	14 (45.2%)	1 (20%)
	University Degree	15 (48.4%)	4 (80%)
	no other education levels ticked		
Work type	apprentice / trainee	1 (3.2%)	
	employee / officer	7 (22.6%)	1 (20%)
	self-employed / freelancer	3 (9.7%)	1 (20%)
	student	20 (64.5%)	3 (60%)
	no other work type ticked		
Employment (%)	fulltime	13 (41.9%)	1 (20%)

	part-time, *M (SD)*	13 (41.9%), 35 (24.66)	3 (60%)
	non-working	4 (12.9%)	1 (20%)
	other	1 (3.2%)	
	other: freelancer		
Income (CHF / month)	up to 2000	10/25 (40%)	NA
	2000 - 3000	6/25 (24 %)	
	3000 - 4000	2/25 (8%)	
	4000 - 5000	1/25 (4%)	
	5000 - 6000	3/25 (12%)	
	6000 - 7000	2/25 (8%)	
	7000 - 80000	0	
	more than 8000	1/25 (4%)	
Marital status	single	29 (93.5%)	5 (100%)
	partnership	2 (6.5%)	
	no other status ticked		
Living situation	alone	3 (9.7%)	5 (100%)
	with partner and/or children	2 (6.5%)	
	living community (excl. family)	17 (54.8%)	
	other	9 (29.5%)	
	other: family of origin (and/or siblings)		

Note. Characteristics of the participants sample and dropouts. Where information is not available for all participants, n or NA is specified.

Supplementary Table 2

Sample description: Motivation to participate (multiple choice)

for intellectual or creative inspiration	13/25 (52%)
gaining insight into myself or parts of my life / self knowledge	19/25 (76%)
curiosity and adventurousness	21/25 (84%)
a general interest in psychedelic medicine or therapy	22/25 (88%)
a general interest in science	15/25 (60%)
simply because of the experience	9/25 (36%)
for more spiritual awareness, understanding or connection	13/25 (52%)
experience the visual effects	5/25 (20%)
someone else suggested/encouraged	1/25 (4%)
a sense of being called by dreams, synchronous events, etc.	1/25 (4%)
want to be high	2/25 (8%)
earn money	2/25 (8%)

Note. N = 31 participants, 6 dropouts. Motivation to participate variables are only available for N = 25 participants.

Supplementary Table 3

Sample description: Drug pre-experience

Drug experience	tobacco	9/31 (29.5%)
	alcohol (mean per month)	28/31 (90.3%) (9.9 p.m.)
	caffeine	22/31 (71%)
within the last 3 months	THC	17/31 (54.8%)
	MDMA	2/31 (6.5%)
	Psilocybin / Magic mushrooms	3/31 (9.7%)
	LSD	2/31 (6.5%)
	Cocaine	1/31 (3.2%)
	Amphetamine / Methamphetamine	1/31 (3.2%)
	Ketamine	1/31 (3.2%)
	Opiates (heroine, morphium)	1/31 (3.2%)
	GHB	1/31 (3.2%)
	others	1/31 (3.2%)
in the past, more than 3 months ago	THC	31/31 (100%)
	MDMA	2/31 (6.5%)
	Psilocybin / Magic mushrooms	11/31 (35.5%)
	LSD	13/31 (41.9%)
	Cocaine	4/31 (12.9%)
	Amphetamine / Methamphetamine	6/31 (19.4%)
	Ketamine	1/31 (3.2%)
	Opiates (heroine, morphium)	1/31 (3.2%)
	GHB	1/31 (3.2%)
	others	1/31 (3.2%)

Note. $N = 31$ participants. Percentage of participants with pre-experience with the respective drugs. A detailed drug pre-experience overview can be found in Supplementary Table 7.

Supplementary Table 4

Participant's detailed drug pre-experience

	min	max	mode	median	mean	SD
THC	0.3	168	4	6	26.4	39.45
MDMA	0	10	0	0	1.71	2.98
Psilocybin / Magic mushrooms	0	15	0	0	1.23	3.17
LSD	0	10	0	0	1.29	2.32
Cocaine	0	7	0	0	0.48	1.55
Amphetamine / Methamphetamine	0	10	0	0	0.48	1.81
Ketamine	0	1	0	0	0.03	0.18
Opiates (heroine, morphium)	0	2	0	0	0.06	0.36
GHB	0	3	0	0	0.1	0.54
Mescaline	0	1	0	0	0.03	0.18
2CB	0	1	0	0	0.06	0.25
Laughing gas	0	1	0	0	0.06	0.25
Ritaline	0	1	0	0	0.1	0.53

Note. N = 31 participants, 6 dropouts. All parameters are given in times / total_lifespan, except for THC in times / year. min = minimum, max = maximum, SD = standard deviation.

Supplementary Table 5

Effects of the Drug Conditions on Subjective Experience and Persisting Effects. N=31. DMT+HAR = DMT+harmine; HAR = harmine only; PLA = placebo. 5D-ASC = 5 dimensions of altered states of consciousness; EBI = emotional breakthrough inventory; CEQ = challenging experience questionnaire; PIQ = psychological insights questionnaire (AMP = avoidance and maladaptive patterns; GAP = goal and adaptive patterns); PEQ = persisting effects questionnaire. Effect sizes: Kendall's W (Friedman's test) and r (Wilcoxon test). ASC, EBI, CEQ: Friedman and Wilcoxon tests. Multiple comparison contrasts with Benjamini-Hochberg correction. PIQ: Mixed model Mixed Model Anova Table (Type 3 tests, S-method) and estimated marginal means (EMMs; degrees-of-freedom method: asymptotic. P value adjustment: Tukey method for comparing a family of 3 estimates).

Subjective experience (retrospective assessment) by drug condition

Scale	subscale	drug condition main effect		contrasts DMT+HAR – HAR		DMT+HAR – PLA		HAR – PLA	
		F(Df)	*p*	*EMM*	*p adj.*	*EMM*	*p adj.*	*EMM*	*p adj.*
5D-ASC	global score asc	80.206 (2,60)	<.001	25.55	<.001	26.29	<.001	0.74	0.95
	experience of unity	42.144 (2,60)	<.001	32.75	<.001	33.88	<.001	1.14	0.96
	spiritual experience	26.349 (2,60)	<.001	24.87	<.001	25.41	<.001	0.54	0.99
	blissful state	60.48 (2,60)	<.001	42.20	<.001	44.20	<.001	2.00	0.898
	insightfulness	41.28 (2,60)	<.001	36.25	<.001	36.51	<.001	0.26	0.998
	disembodiment	38.84 (2,60)	<.001	34.26	<.001	36.20	<.001	1.95	0.91
	impaired cognition and control	28.52 (2,60)	<.001	19.38	<.001	20.12	<.001	0.73	0.97
	anxiety	9.91 (2,60)	<.001	4.24	0.001	4.53	0.000	0.29	0.96
	elemenatry imagery	175.98 (2,60)	<.001	57.77	<.001	59.66	<.001	1.88	0.86
	complex imagery	87.32 (2,60)	<.001	55.90	<.001	55.59	<.001	-0.31	0.998
	audio visual synesthesiae	38.42 (2,60)	<.001	33.35	<.001	35.38	<.001	2.02	0.896
	changed meaning of percepts	45.43 (2,60)	<.001	33.43	<.001	32.50	<.001	-0.94	0.97
PIQ	global score	28.51 (2, 58.5)	<.001	1.12	<.001	1.278	<.001	0.155	.687
	AMP	22.49 (2, 58.5)	<.001	0.98	<.001	1.093	<.001	0.110	.816
	GAP	32.59 (2, 58.5)	<.001	1.26	<.001	1.478	<.001	0.223	.506
EBI	emotional breakthrough	26.51 (2,60)	<.001	24.49	<.001	25.78	<.001	1.28	.94
CEQ	global score	12.84 (2, 60)	<.001	4.48	0.0001	5.13	<.0001	0.648	.83
	fear	5.09	.009	4.65	0.0307	5.42	.0089	0.774	.91
	grief	4.28	.018	4.95	0.0408	5.38	.0230	0.430	.98
	physical distress	14.01	<.001	8.13	0.0007	11.35	<.0001	3.230	.31
	insanity	7.86	<.001	4.95	0.0013	4.73	.0023	-0.215	.99
	isolation	1.86	.165	5.16	0.2613	5.81	.1834	0.645	.98
	death	3.94	.023	1.94	0.0399	1.94	.0399	0.000	1.0
	paranoia	1.97	.149	1.61	0.1459	1.29	.2909	-0.323	.93

Supplementary Table 6

Development of Psychopathology Symptoms Measured with the Symptom Checklist (SCL-90) from Baseline to Follow-up and Persisting Effects. DMT+HAR = DMT+harmine; HAR = harmine only; PLA = placebo. SCL-90-R: Friedman test and Dunn-Bonferroni test for multiple comparison of groups. Contrasts are shown between the drug conditions for the experiential variables, and between the assessment timepoints for the baseline–follow-up changes (SQL-90-R). *Z*-values represent effect sizes

of Dunn-Bonferroni tests. Baseline–follow-up changes in psychopathology (SCL-90-R) concern the full study participation (all drug conditions). Only full datasets were analyzed (participants with all three timepoints and all subscales), *n* = 22. PEQ: Wilcoxon rank sum test with continuity correction, effect size *r*. 1m = 1 month, 4m = 4 months. Persisting effects concern the full study participation (all drug conditions), *n* = 31

Baseline – follow up changes									
				contrasts					
Scale	subscale	timepoint main effect		baseline – follow up 1 m		baseline – follow up 4 m		follow up 1 m – 4 m	
		χ2(df2)	*p*	*z*	*p adj.*	*z*	*p adj.*	*z*	*p adj.*
SCL-90-R	somatization	3.66	.161	-0.31	1.000	-0.35	1.000	-0.04	.968
	obsessive compulsion	9.18	.010	2.60	.028	2.00	.068	-0.59	.554
	interpersonal sensitivity	11.41	.003	1.89	.178	1.58	.170	-0.30	.763
	depression	4.03	.134	1.89	.178	1.58	.170	-0.30	.763
	anxiety	6.78	.034	1.82	.103	1.85	.191	0.03	.973
	hostility	2.26	.323	1.18	.355	0.00	1.000	-1.18	.710
	phobic anxiety	2.96	.228	1.43	.460	0.33	.744	-1.10	.406
	paranoid ideation	5.88	.053	2.42	.046	1.50	.201	-0.93	.355
	psychoticism	6.65	.036	1.74	.245	0.52	.603	-1.22	.333
Persisting effects at 1 and 4 months follow-up, comparison positive vs. negative persisting effects									
				positive vs. negative (both follow ups)		positive vs. negative follow up 1 m		positive vs. negative follow up 4 m	
				r	*p*	*r*	*p*	*r*	*p*
PEQ	attitude			0.83	<.001	0.91	<.001	0.79	<.001
	mood			0.57	<.001	0.57	.004	0.05	.003
	altruistic / social			0.46	.001	0.57	.004	0.39	.051
	behavior			0.78	<.001	0.77	<.001	0.83	<.001

Supplementary Figure 1

5D-ASC spider plots

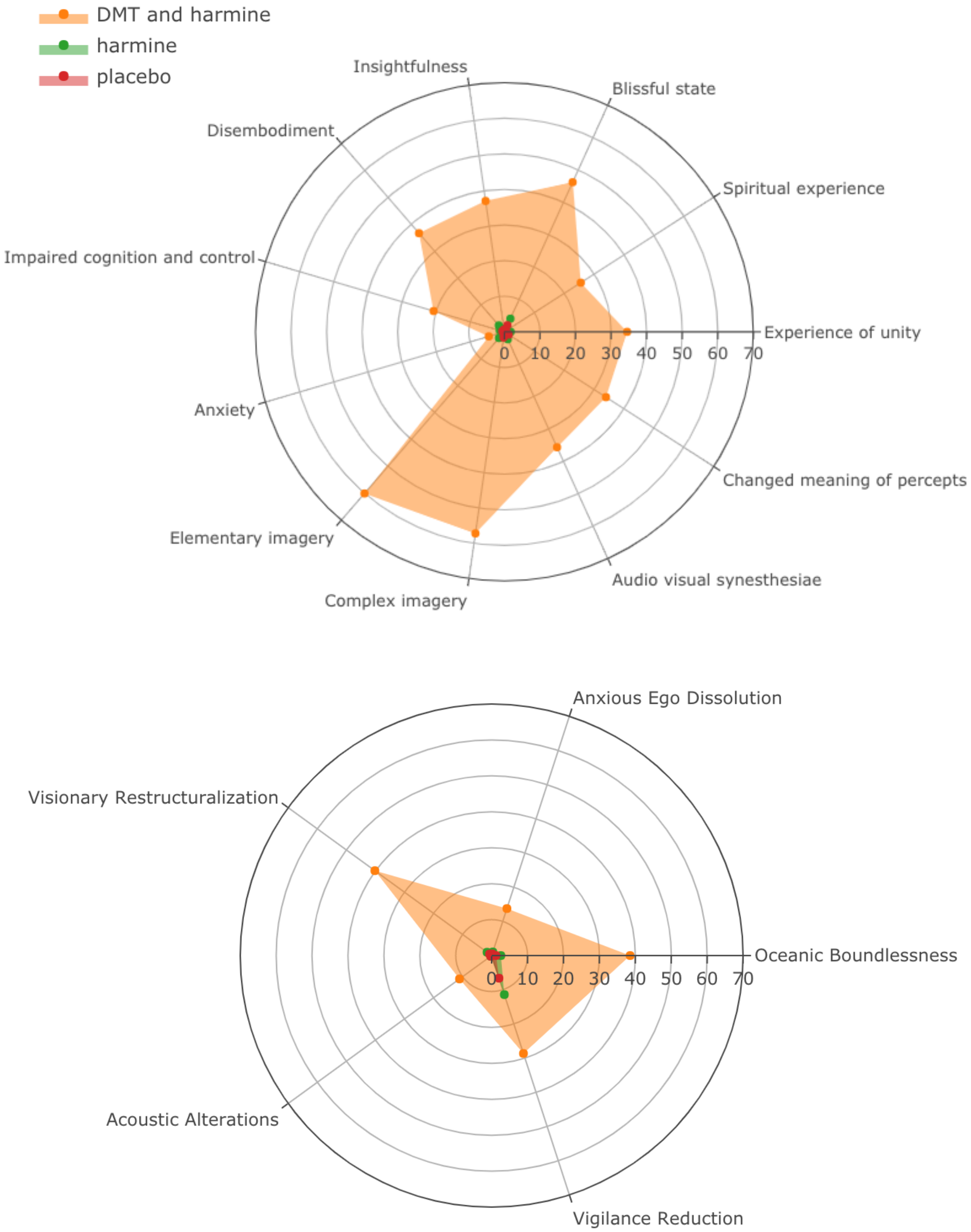

Note. Spider plots of 5D-ASC (5 dimensions and 11 subscales) are shown for better comparability with other trials using this type of visualization. Because of the small levels for harmine only and placebo, boxplots (drug conditions next to each other) are shown in the main article.

Supplementary Figure 2

Significance ratings at the 4 months follow-up

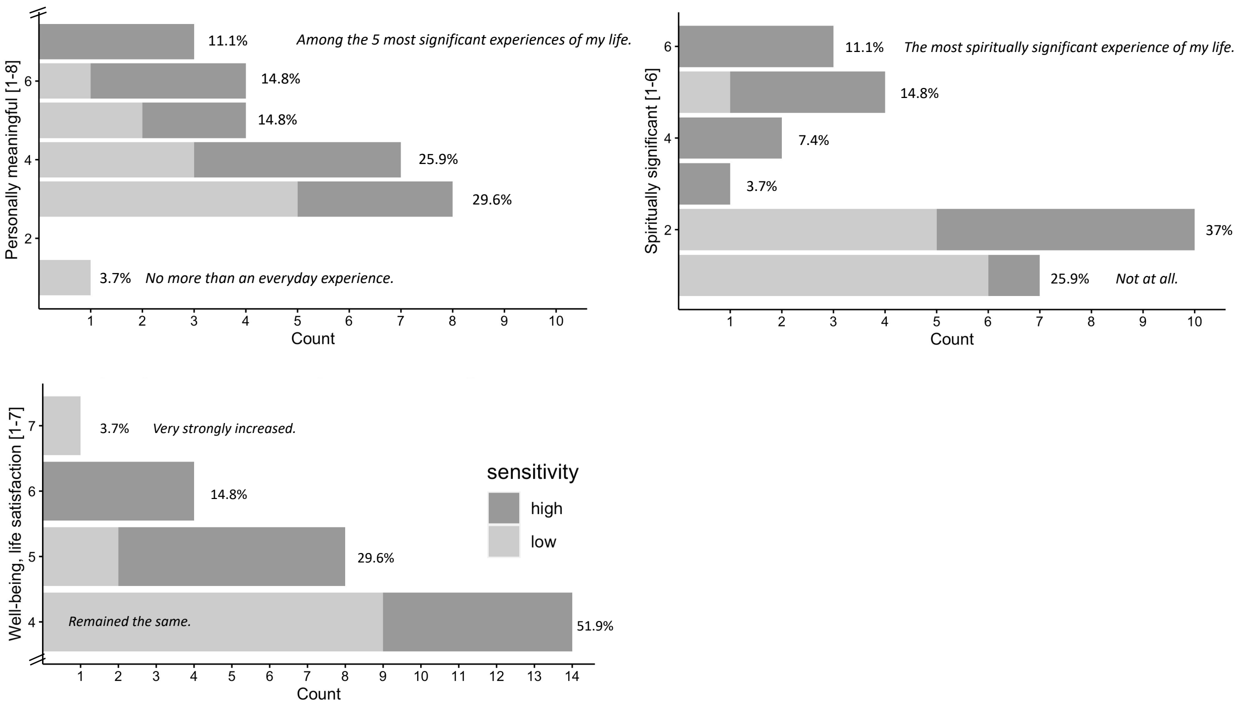

Note. N=27. Significance ratings at the 4 months follow up. In the main paper, significance ratings at the 1 month follow up are shown. A) How personally meaningful was the experience? B) Indicate the degree to which the experience was spiritually significant to you. C) Do you believe that the experience and your contemplation of it have led to a change in your current sense of personal well-being or life satisfaction?

Supplementary Figure 3

Trait measures at baseline, follow-up 1 month and 4 months

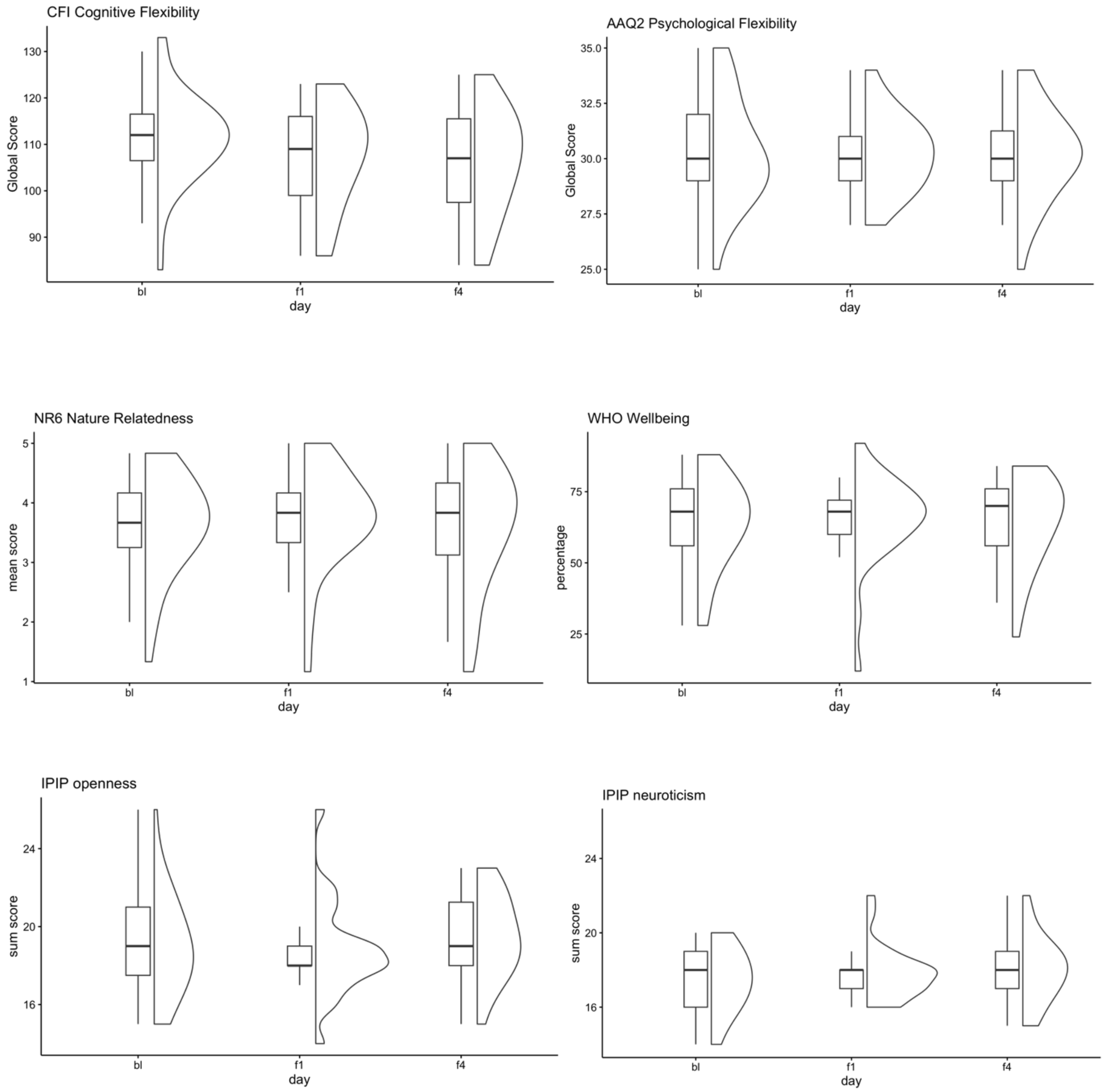

Supplementary: Qualitative Diary Reports

Therapy-relevant Psychological Processes

"I notice how I try to look at emotions and thoughts from a greater distance without judging them. It is difficult, but since the trip one of my goals. I also try to build a greater acceptance for thoughts and decisions and to go through life with what is and not what is not. During the trip I had the strongest feeling ever that everything is ok the way it is."

"I lived completely in the moment. Perhaps this is a lesson that has been confirmed once again: All that matters is the here and now. Live in the moment!"

"...a great calm spread through me, and it felt like everything was settled and I could continue with what I like to do or where I was drawn to. My satisfaction and connection to my intuition is very great at the moment."

"Today I noticed a greater appreciation for everything. I was very aware of my thoughts, feelings & actions and tried to carry them out as lovingly as possible. I did everything I set out to do with joy & passion."

Psychological Safety and Tolerability

"When I closed my eyes, I could dive into worlds and be really absorbed by that, but as soon as I opened my eyes I could focus on the now again. Talking and interacting with people went very well..."

Persisting Effects, Integration, and Contextual Factors

"The rest of the evening I felt very thoughtful, but in a positive sense. There was just a lot to process and think about again. On the one hand what I experienced, but also how I perceived it and what I can now do with it in everyday life."

"... difficult to implement everything learned and not to fall back into the same patterns. I have the feeling that you need several such trips and states to be able to really change that sustainably."

Limitations

"*I think that the whole setting and the tasks have/had a big effect, at least on me, that I probably could not get so involved in the profound questions. The many changes of persons*

and tasks were probably a contributing reason that I did not have an unpleasant feeling, but a feeling of not completely surrendering to the mind's eye and what was happening \'within me\'."

"In this setting, it was difficult for me to benefit optimally from the experience. I could never fully immerse myself in the experience. I experienced a lot of interesting thoughts & insights and experiences and in the next moment I was pulled out again by the experimental setting. This was to be expected and even though I would have liked to dive deeper into the experience to take more away from it, I realize that this was not the time & place for that. I was here in the context of a scientific investigation & trying my best to comply. To benefit optimally from the experience it would probably have to be built into a psychotherapeutic context."
